# Interplay between SARS‐CoV‐2‐derived miRNAs, immune system, vitamin D pathway and respiratory system

**DOI:** 10.1111/jcmm.16694

**Published:** 2021-06-22

**Authors:** Elham Karimi, Hanieh Azari, Maryam Yari, Ahmad Tahmasebi, Mehdi Hassani Azad, Pegah Mousavi

**Affiliations:** ^1^ Student Research Committee Faculty of Medicine Hormozgan University of Medical Sciences Bandar Abbas Iran; ^2^ Department of Medical Biotechnology School of Advanced Medical Sciences and Technologies Shiraz University of Medical Sciences Shiraz Iran; ^3^ Institute of Biotechnology Shiraz University Shiraz Iran; ^4^ Infectious and Tropical Diseases Research Center Hormozgan Health Institute Hormozgan University of Medical Sciences Bandar Abbas Iran; ^5^ Department of Medical Genetics Faculty of Medicine Hormozgan University of Medical Sciences Bandar Abbas Iran

**Keywords:** bioinformatics, immune system, miRNA, respiratory system, SARS‐COV2, vitamin D pathway

## Abstract

The new coronavirus pandemic started in China in 2019. The intensity of the disease can range from mild to severe, leading to death in many cases. Despite extensive research in this area, the exact molecular nature of virus is not fully recognized; however, according to pieces of evidence, one of the mechanisms of virus pathogenesis is through the function of viral miRNAs. So, we hypothesized that SARS‐CoV‐2 pathogenesis may be due to targeting important genes in the host with its miRNAs, which involved in the respiratory system, immune pathways and vitamin D pathways, thus possibly contributing to disease progression and virus survival. Potential miRNA precursors and mature miRNA were predicted and confirmed based on the virus genome. The next step was to predict and identify their target genes and perform functional enrichment analysis to recognize the biological processes connected with these genes in the three pathways mentioned above through several comprehensive databases. Finally, cis‐acting regulatory elements in 5′ regulatory regions were analysed, and the analysis of available RNAseq data determined the expression level of genes. We revealed that thirty‐nine mature miRNAs could theoretically derive from the SARS‐CoV‐2 genome. Functional enrichment analysis elucidated three highlighted pathways involved in SARS‐CoV‐2 pathogenesis: vitamin D, immune system and respiratory system. Our finding highlighted genes' involvement in three crucial molecular pathways and may help develop new therapeutic targets related to SARS‐CoV‐2.

## 1 INTRODUCTION

The first case of coronavirus disease (severe acute respiratory syndrome coronavirus 2 (SARS‐CoV‐2)) in human was reported in late December 2019 in Wuhan, China, which has subsequently affected all over the world. On 11 March 2020, according to the World Health Organization (WHO), SARS‐CoV‐2 was introduced as a pandemic, which is the third epidemic caused by coronaviruses in the past 20 years.^1^

SARS‐CoV‐2 (also known as coronavirus disease 2019 (COVID‐19)) is a positive‐stranded enveloped RNA virus. It has a +ssRNA genome with nearly 30 kb of size (ranging from 26 000 to 37 000 bases) in length. Genomic structure of SARS‐CoV‐2 comprises of a 5′‐leader‐untranslated region(UTR)‐replicase‐S (Spike)‐E (Envelope)‐M (Membrane)‐N (Nucleocapsid)‐3′UTRpoly (A) tail.^2^ Each part of SARS‐CoV‐2 has a unique role in viral pathogenesis, and isolation of SARS‐CoV‐2 genomic sequence has demonstrated 88% of identity with two bat‐derived SARS‐like coronaviruses.^3^

Immune system is the first and currently the only defence against the virus. Once, the virus enters the body and is detected by the angiotensin‐converting enzyme 2(ACE2) factor, the virus enters the host cell and inserts its RNA genome into cytoplasm of that cell. There, it multiplies and a large number of new viral particles germinate out of the host cell and move to other cells. In the first stage, the immune system responds by releasing a large number of inflammatory cytokines, such as interleukin 8(IL‐8), eventually activating T lymphocytes and neutrophils and then uses the acquired immune system alongside innate immune system.^4^ This results in activation of a vast range of signalling pathways, such as Janus kinase (JAK)/signal transducer and activator of transcription (STAT) and nuclear factor kappa B (NF/κB).^5^ In this case, if pathogenicity of the virus continues and more inflammatory cytokines are produced, the immune system activity can lead to serious tissue damage and even tissue death. Therefore, the immune system is one of the most challenging cases in the field of pathogenicity of this virus, which needs to be studied extensively.^6^ Another issue in pathogenesis of the virus is the influence of vitamin D on its prevention. Many studies have demonstrated that the decreased levels of vitamin D are associated with the increased disease severity and mortality, and vitamin D supplements are widely recommended during the pandemic period.^7^, ^8^, ^9^ The symptoms of SARS‐CoV‐2 are different from mild to severe. Investigations have shown that the patients with COVID‐19 infection have low lymphocyte counts, unusual respiratory results and elevated levels of pro‐inflammatory cytokines in plasma.^10^, ^11^

MicroRNAs play a role in important biological processes, such as cellular metabolism, cell division, death, cell movement, intracellular signalling, immunity, apoptosis and oncogenesis.^12^ They can interact with different regions of target mRNA including 5‐UTR, gene promoter and coding sequences. Binding with 3‐UTR causes post‐transcriptional silencing rather than pairing with 5'‐UTR or coding sequence, while miRNA interaction with promoter regions has been proved to induce transcription.^13^ Viruses can also produce miRNAs in their genomes that can target host genes. To date, more than 250 new viral miRNAs have been discovered, making it possible to detect function and origin of virus‐encoded miRNAs. However, a large proportion of these miRNAs belong to DNA virus, and only 30 mature miRNAs to RNA viruses have been identified.^14^ Canonical biogenesis of miRNA is a multi‐step process involving transcription of viral miRNA gene by RNA pol II and formation of a structure called as pri‐miRNA, its breakdown by Drosha endonuclease and DiGeorge syndrome critical region 8 (DGCR8) complex in the form of a hairpin structure called as pre‐miRNA, its extraction from the nucleus and maturation through Dicer endonuclease, eventually joining the RNA‐induced silencing complex (RISC) and suppressing expression of host genes.^15^ Cell penetration by SARS‐CoV‐2 is mediated by membrane fusion process, in which a +ssRNA‐sense genome is delivered into the cytoplasm, serving as a template for virus replication process utilizing virally encoded RNA‐dependent RNA polymerase (RdRp), and ultimately, protein synthesis in fact, miRNA biogenesis process in cytoplasmic viruses is performed by non‐canonical miRNA biogenesis pathways in Drosha‐ or Dicer‐independent manner.^16^, ^17^ It has recently been shown that one of the main mechanisms of virus pathogenesis is targeting key human genes and suppressing their expression through viral miRNAs.^18^ Therefore, study of viral microRNAs allows us to become more familiar with mechanism of coronavirus pathogenesis. In fact, viral microRNAs play a role in completing virus life cycle. In this way, they try to maintain their survival and proliferation of their genomes by targeting genes of the immune system. For example, herpes simplex virus type 1(HSV1), Kaposi҆ sarcoma–associated herpesvirus (KSHV) and human cytomegalovirus (HCMV) encode DNA viruses that regulate viral genes or host genes involved in latent and persistent infection.^19^ Human immunodeficiency virus 1 (HIV‐1), bovine leukaemia virus, bovine foamy virus, avian leucosis virus, dengue virus and Ebola virus are examples of RNA viruses producing miRNAs that target host genes.^20^, ^21^, ^22^, ^23^, ^24^, ^25^

**FIGURE 1:**
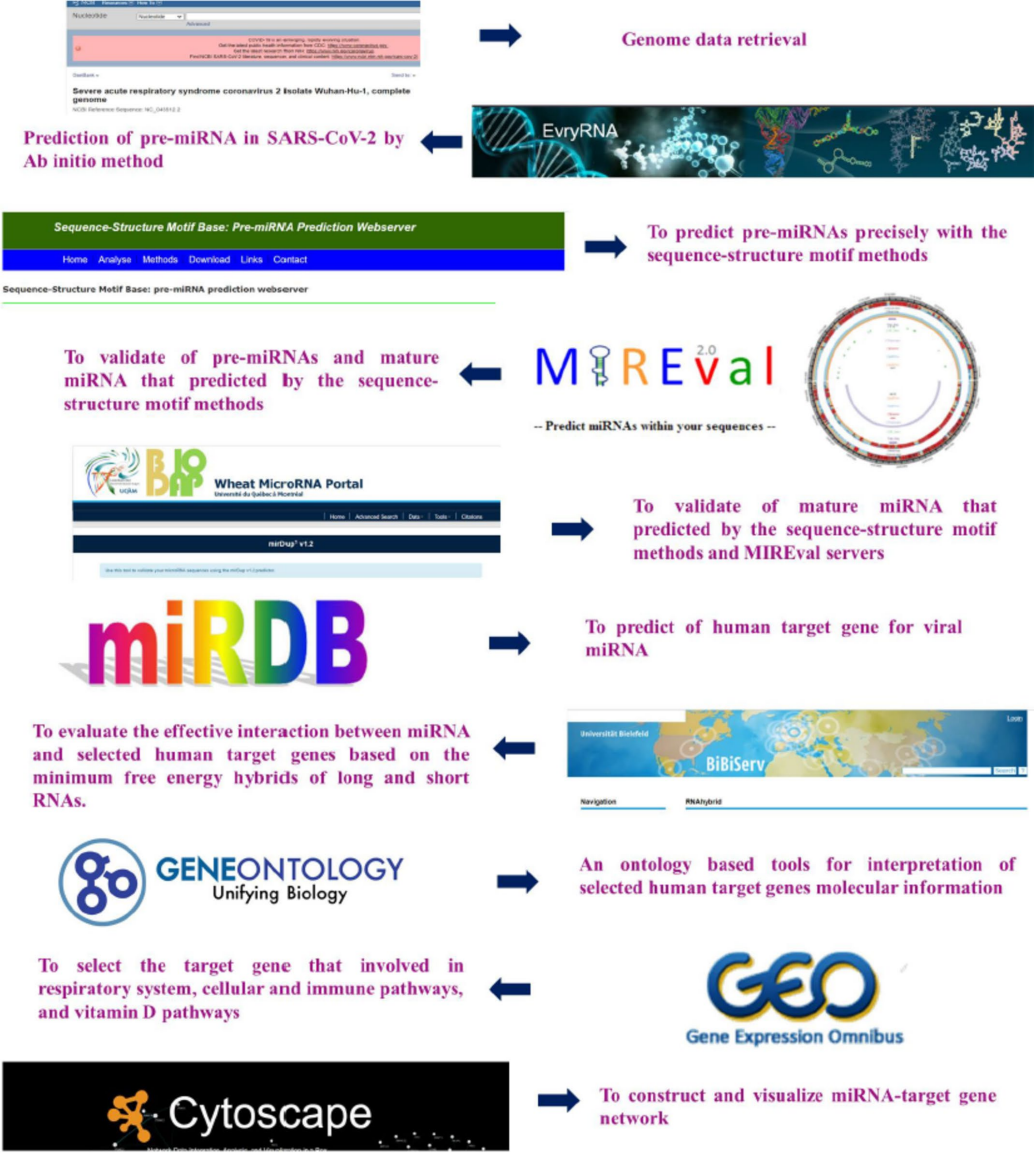
Flow chart of the methodology used in the study

Using bioinformatics approach, herein, novel SARS‐CoV‐2 encoded miRNAs were identified. Our findings demonstrated that SARS‐CoV‐2 miRNAs probably have putative role in virus pathogenesis and affect the host immune system and various physiological processes to take advantage of the prolonged refuge in host cell. It was found that SARS‐CoV‐2 miRNA–targeted human host genes are involved in viral pathogenesis, such as respiratory system, cellular and immune pathways, and vitamin D pathways. Finding target genes of these miRNAs and their pathways could provide new insights into SARS‐CoV‐2 infection, pathogenesis and treatment design also; enrichment analysis of each of the host's target genes provides more information about the SARS‐CoV‐2 (Figure 1).

## 2 MATERIALS AND METHODS

### 2.1 Retrieval of genome data

FASTA format of the virus genome sequence was acquired from the National Center for Biological Information (NCBI) available at (https://www.ncbi.nlm.nih.gov/ ) using accession no. NC_045512.2.^26^

### 2.2 Identification of microRNA precursor

For prediction of miRNA precursors, the miRNAFold web database (https://evryrna.ibisc.univ‐evry.fr/miRNAFold) was used with default parameters (Sliding window size: 150, Percentage of verified features: 70). As a result, the pre‐miRNAs' sequence and positions were obtained.^27^

### 2.3 Prediction of potential pre‐miRNAs and mature miRNAs

One of the most critical steps in predicting mature miRNA is selecting bioinformatics tools that could differentiate between two miRNA precursors, pre‐miRNA and pri‐miRNA. pri‐miRNA, the precursor of pre‐miRNAs, is structurally characterized by a terminal loop, a stem of approximately three helical turns that flanked by two basal unpaired sequences.^28^ Sequence‐Structure Motif Base (http://www.regulatoryrna.org/webserver/SSMB/pre‐miRNA/index.html), a pre‐miRNA prediction web server, was used to differentiate between pri‐miRNA and pre‐miRNAs and predict mature miRNAs between a complex of the conserved stem‐loop structures.^29^

It utilizes effective PriMir and Mirident programs, accurately approved to predict pre‐miRNA and RNAfold software to plot its secondary structure. This resource consists of a set of Perl, Python and PHP programs.

Besides, miREval 2.0, a web server available at http://mimirna.centenary.org.au/mireval/ , was applied to further validate resulting pri‐miRNAs. It uses support vector machines (SVMs) as a useful machine learning method for predicting accurate miRNAs.^30^

The least bases for the stem‐loop were set to 22 for the Sequence‐Structure Motif Base.

Finally, miRdup v1.2, an online prediction server is accessible at (http://wheat.bioinfo.uqam.ca./index.php?action=mirdup), which uses a random forest classifier working based on experimentally validated miRNAs for further validation of mature miRNA sequence in pre‐miRNA using their default parameters.^31^

### 2.4 Obtaining SARS‐CoV‐2 miRNA target genes

The miRDB database (http://mirdb.org/) was implemented to identify the targeted genes by the predicted miRNAs with score 80 as a cut‐off.^32^

Human genes that are related to SARS‐CoV‐2 infection were extracted from the gene ontology database (http://geneontology.org/).^33^ Subsequently, a list of hub genes targeted by these miRNAs was identified followed by running the overlap analysis between miRNA target genes and corresponding human genes in SARS‐CoV‐2 infection using the Venn diagram (http://bioinformatics.psb.ugent.be/webtools/Venn/).

A principal hypothesis regarding miRNA/target functional interaction is a conserved base pairing (Watson‐Creek match) between miRNA and target at miRNA positions 2‐7 or 8 (5´ upstream of miRNA).^34^ Recognizing and confirming interplays between miRNAs and their downstream targets is essential in describing regulative capacity of miRNAs in complex systems controlling biological processes.^35^ RNAhybrid program (https://bibiserv.cebitec.uni bielefeld.de/rnahybrid/), which can determine the possible binding sites of multiple miRNAs in large target RNAs based on the minimum free energy of hybrid structure, was performed for reliable prediction of the most reliable energy hybridization of v‐miRNAs and 3´UTR of their target mRNAs.^36^ The higher the degree of complementarity between the seed region sequence in miRNA and the 3 UTR in the mRNA target gene, the more reliable the prediction, so according to the other previous studies, the minimum six‐base seed match length was considered. For this evaluation, input data, that is 3 UTR of host candidate target genes (extracted from the miRDB database) and mature miRNA, were uploaded.

### 2.5 Analysis of gene, function and pathway enrichment

For gaining mechanistic insight into our gene list, gene ontology (GO), Kyoto Encyclopedia of Genes and Genomes (KEGG) (KEGG; https://www.genome.jp/kegg/) and over‐representation analyses were conducted on the list of target genes using the R software cluster profiler package.^37^ Significant groups were determined according to Benjamini‐Hochberg correction and the adjusted cut‐off levels of <0.05. In addition, Cytoscape tool (version 3.8.0) was used to create and visualize a miRNA‐target gene network.

### 2.6 Expression analysis

Gene expression omnibus (GEO), an organized database for functional genomic studies, is a free source of the results regarding microarray expression gene stored at NCBI (https://www.ncbi.nlm.nih.gov/geo). Corresponding GEO entries for evaluating expression level of each of the target genes were RNA‐seq transcriptomic expression data set GSE148829 of SARS‐CoV‐2‐infected human samples using the platform GPL18573 Illumina NextSeq 500 (Homo sapiens), GSM4462413 and GSM4462414 samples (as a control sample), and GSM4462415 and GSM4462416 samples (as the infected patients’ sample).

### 2.7 Cis‐acting regulatory elements in 5′ regulatory regions

Investigation of cis‐acting regulatory elements (CREs) can provide useful information on transcriptional regulation of the genes involved in response to SARS‐CoV‐2 infection; hence, the 1000 bp was retrieved relative to transcriptional start sites of target genes from the human Ensembl genome browser (http://human.ensembl.org). MEME, a motif discovery algorithm publicly available (meme.nbcr.net/meme/intro.html) (Bailey et al, 2009), was implemented to identify and characterize the conserved cis‐motifs of transcription on DNA sequences with its default parameters excluding for the maximum number of motifs (11) with a threshold E‐value of <1e‐4. CREs related to the target genes were compared with the known candidate motifs presented in the JASPAR database 2018 release with a threshold E‐value cut‐off of 0.05 using the TomTom v 5.0.1 tool (https: // meme suite.org/tools/tomtom).^38^ GoMo tool, Gene Ontology Motif Enrichment (http://meme‐suite.org/ tools/gomo) and UniProtKB database (https://www.uniprot.org/uniprot/O60765) were employed to distinguish the potential biological process of each identified motif in promoter region.^39^

## 3 RESULTS

### 3.1 Potential miRNA, pre‐miRNAs and mature miRNAs

Viral miRNA is a type of miRNA recently discovered and expressed to change host cell behaviour and gene expression by inducing degradation, translation, inhibition or other mechanisms, as well as host cell function. The COVID‐19 viral genome is a single‐strand RNA molecule with linear topology, consisting of 29,903 nucleotide base pairs. Totally, 519 putative pre‐miRNAs were detected by the miRNAFold web server among the whole genome. For finding accurate mature miRNAs from their pre‐miRNAs and precursors, several different bioinformatics tools were applied. The Sequence‐Structure Motif Base server was used, and 147 pre‐miRNAs were verified and 309 mature miRNAs were predicted through a set of conserved stem‐loop structures. For further reassurance, 147 pre‐miRNAs previously introduced by Motif Base were analysed by miREval 2.0 tool and 106 pre‐miRNAs were approved.

Finally, the results including partial evaluation, the predicted hairpins' dot‐bracket secondary structure, free energy and sequence composition were conceptualized by SVM method using Verna v3.9 tool (the Varna GUI) (http://varna‐gui.software.informer.com/) and were aligned with respect to the known miRNAs.

For accurately validating and checking mature miRNAs detected by miREval tool, the miRdup online tool was employed that recognized 39 mature miRNAs in the final step (Table 1) and provided detailed results (supplementary Data file 1).

**TABLE 1:** Thirty nine mature miRNAs recognized by miRdup database and their targeted host genes

Name	Sequence	Target genes	Name	Sequence	Target genes
SCoV‐2‐miR‐1	UUGGCUACUAACAAUCUAGUUG	GGCX, PRRC2B	SCoV‐2‐miR‐21	UUGGGUAGUGCUUUAUUAGAAG	ARL6IP6, TBCA
SCoV‐2‐miR‐2	GAAAUACCAGUGGCUUACCGCA	FBN2, ZNF503, RAB2A	SCoV‐2‐miR‐22	AAGCUAAAAGACUGUGUUAUGU	ATE1, RAB10, RDX, MARK2
SCoV‐2‐miR‐3	CGCGACGUGCUCGUACGUGGCU	_	SCoV‐2‐miR‐23	GGUACAACAUUUACUUAUGCAU	MARK1
SCoV‐2‐miR‐4	CGCGACGUGCUCGUACGUGGCU	_	SCoV‐2‐miR‐24	UCGUGUUGUCUGUACUGCCGUU	_
SCoV‐2‐miR‐5	GGCUACUAACAAUCUAGUUGUA	ITGB1	SCoV‐2‐miR‐25	CAGGGCUUUAACUGCAGAGUCA	MARK1, TMED5
SCoV‐2‐miR‐6	AAACUCAAACCCGUCCUUGAUU	PTBP2	SCoV‐2‐miR‐26	UCACAUGUUGACACUGACUUAA	
SCoV‐2‐miR‐7	UCUGCCUAUACAGUUGAACUCG	HYOU1, EDEM3, PLAT	SCoV‐2‐miR‐27	AGUCAUCGUCAACAACCUAGAC	DDX21, ALG5
SCoV‐2‐miR‐8	UUGUGGCAGAUGCUGUCAUAAA	SEPSECS, PRKAR2B	SCoV‐2‐miR‐28	UGAAAUGGUCAUGUGUGGCGGU	_
SCoV‐2‐miR‐9	AAACAAUUGUUGAGGUUCAACC	RALA, RAE1, ZC3H7A, STOML2, PRKAR2B	SCoV‐2‐miR‐29	UGCUCGCAUAGUGUAUACAGCU	_
SCoV‐2‐miR‐10	CUUAUUACAGAGCAAGGGCUGG	_	SCoV‐2‐miR‐30	GCUUAAAGCACAUAAAGACAAA	GNG5
SCoV‐2‐miR‐11	UCUUAGCCUACUGUAAUAAGAC	MYCBP2	SCoV‐2‐miR‐31	UGCACAUGUAGCUAGUUGUGAU	TBCA
SCoV‐2‐miR‐12	GUGCACUUAUCUUAGCCUACUG	_	SCoV‐2‐miR‐32	UGAUCUUUAUAAGCUCAUGGGA	HS2ST1, SBNO1, SPART
SCoV‐2‐miR‐13	UGAGUUAGGUGAUGUUAGAGAA	_	SCoV‐2‐miR‐33	UCAACUGAAAUCUAUCAGGCCG	_
SCoV‐2‐miR‐14	AAGCAUCUAUGCCGACUACUAU	_	SCoV‐2‐miR‐34	CGGCGGGCACGUAGUGUAGCUA	RAB7A
SCoV‐2‐miR‐15	AUCCUACUGACCAGUCUUCUUA	_	SCoV‐2‐miR‐35	CUACACUAUGUCACUUGGUGCA	_
SCoV‐2‐miR‐16	UUGAUAGUGUUACAGUGAAGAA	_	SCoV‐2‐miR‐36	UUAUGCUUUGCUGUAUGAC	_
SCoV‐2‐miR‐17	ACUUUGAUAAAGCUGGUCA	_	SCoV‐2‐miR‐37	CGCAGCGUGUAGCAGGUGACUC	_
SCoV‐2‐miR‐18	UGUAUCUAAAGUUGCGUAGUGA	_	SCoV‐2‐miR‐38	GAAAUACCAGUGG	_
SCoV‐2‐miR‐19	CCCUAAUUAUGAAGAUUUACUC	_	SCoV‐2‐miR‐39	CAUAAUUUCUUGGU	_
SCoV‐2‐miR‐20	UUGUUACAUGCACCAUAUGGAA	_			

### 3.2 Identification of SARS‐CoV‐2 miRNA target genes

**FIGURE 2:**
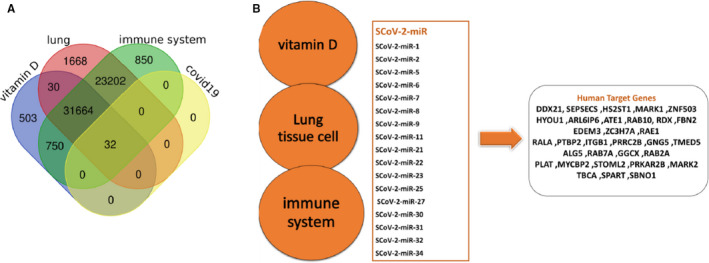
miRNAs and their target gene involvement in three possible pathway of coronavirus pathogenicity including vitamin D, immune system and the respiratory system pathways. A, Venn diagram showing the commonality between pathways. B, Figure indicating detailed miRNAs and their target genes in mentioned pathways

**FIGURE 3:**
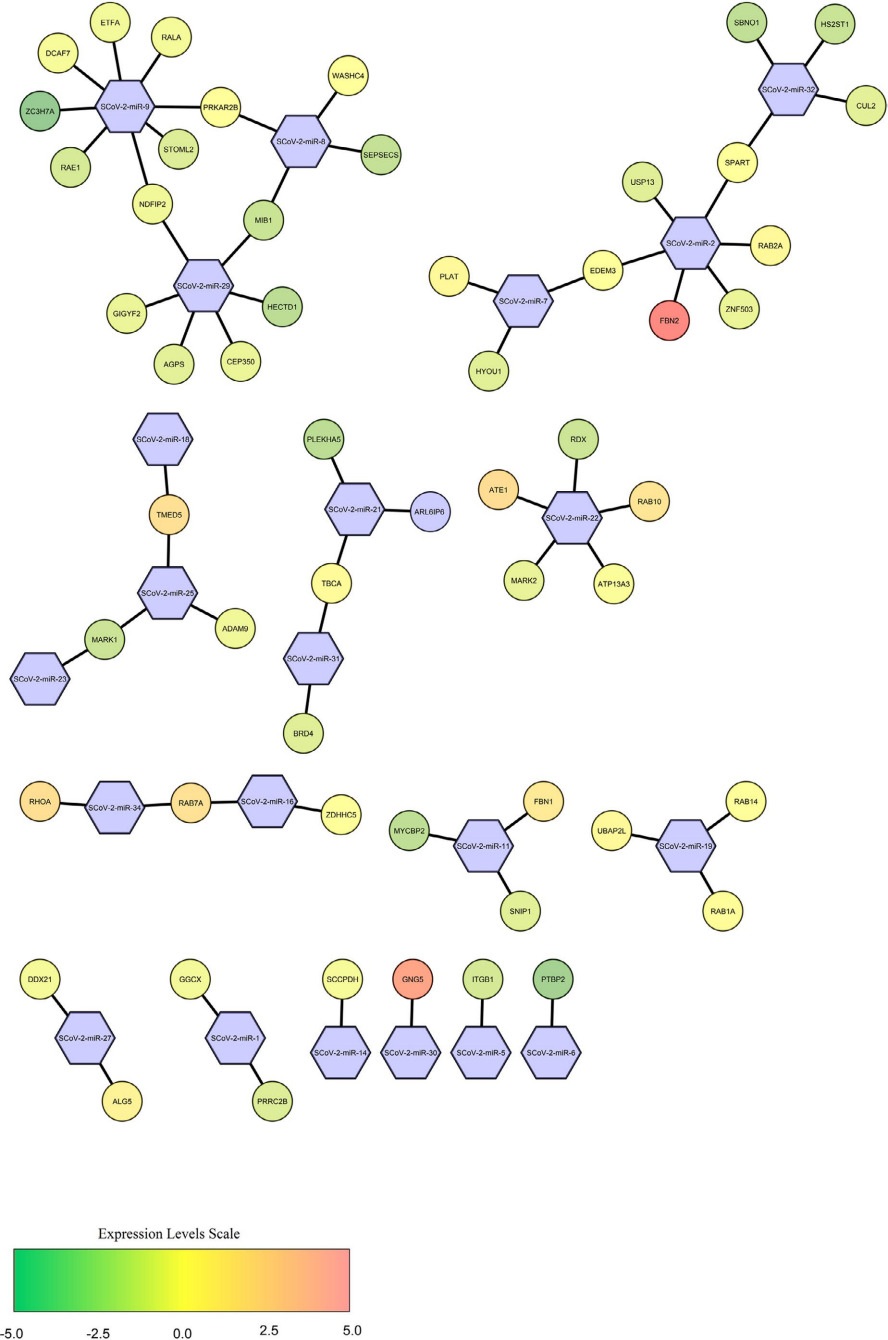
The constructed molecular network SARS‐CoV‐2‐derived miRNAs (circle) with their target human host genes (hexagonal) related to respiratory system, cellular and immune pathways and vitamin D pathways, involved in viral pathogenesis, using Cytoscape version 3.8.0. The genes' transcription levels are described on a colour‐coded scale, where up‐ and down‐regulated molecules are displayed towards red and green, respectively, and yellow signifies a slight change in expression level. miRNAs are shown in coloured blue: SCoV‐2‐miR, SARS‐CoV‐2‐derived microRNA

Herein, a bidirectional analysis was used in order to achieve genes targeted with these miRNAs. In the first step, 5,584 genes targeted by the miRNAs were found with an 80% of cut‐off and they were selected for further analysis. Concurrently, a list of 301 genes predicted to have a role in COVID‐19 was downloaded from a gene ontology database (http://geneontology.org/covid‐19.html) through high‐throughput analysis. The overlapping analysis was performed to see if there is a commonality between target genes and the mentioned data set. Seventy‐five genes were detected and entered the next level. For ensuring about effectiveness of the interactions between miRNAs and the listed target genes, RNAhybrid tool was applied with a cut‐off level described in the Methodology Section (supplementary Data file 2). After removal of duplicates, 32 interactions were considered to be effective based on the cut‐off level mentioned in the Methodology Section. As shown in Table 2, among 39 final miRNAs, only 17 miRNAs had interactions with these target genes. Since vitamin D, the immune system and lung tissue cells are believed to be the target of COVID‐19 infection, the involved genes of each pathway were obtained through GEO under accession numbers of GSE152418, GSE166703 and GSE156124, and Venn diagram was used to see if there were any similarities between RNAhybrid results and these three sets of genes (the results are shown in Tables 1 and 2). According to the results, all 32 genes were common between three considered pathways (Figure 2). The miRNA‐target gene network was also visualized using Cytoscape tool (Figure 3).

**TABLE 2:** The basic information related to host genes targeted by the SARS‐CoV‐2 miRNAs

Gene name	Protein name	Uniport number
*GGCX*	Vitamin K‐dependent gamma‐carboxylase	P38435 (VKGC_HUMAN)
*PRRC2B*	Protein PRRC2B	Q5JSZ5 (PRC2B_HUMAN)
*FBN2*	Fibrillin‐2	P35556 (FBN2_HUMAN)
*ZNF503*	Zinc finger protein 503	Q96F45 (ZN503_HUMAN)
*RAB2A*	Ras‐related protein Rab‐2A	P61019 (RAB2A_HUMAN)
*ITGB1*	Integrin beta‐1	P05556 (ITB1_HUMAN)
*PTBP2*	Polypyrimidine tract‐binding protein 2	Q9UKA9 (PTBP2_HUMAN)
*HYOU1*	Hypoxia up‐regulated protein 1	Q9Y4L1 (HYOU1_HUMAN)
*EDEM3*	ER degradation‐enhancing alpha‐mannosidase‐like protein 3	Q9BZQ6 (EDEM3_HUMAN)
*PLAT*	Tissue‐type plasminogen activator	P00750 (TPA_HUMAN)
*SEPSECS*	O‐phosphoseryl‐tRNA (Sec) selenium transferase	Q9HD40 (SPCS_HUMAN)
*PRKAR2B*	cAMP‐dependent protein kinase type II‐beta regulatory subunit	P31323 (KAP3_HUMAN)
*RALA*	Ras‐related protein Ral‐A	P11233 (RALA_HUMAN)
*RAE1*	mRNA export factor	P78406 (RAE1L_HUMAN)
*ZC3H7A*	Zinc finger CCCH domain‐containing protein 7A	Q8IWR0 (Z3H7A_HUMAN)
*STOML2*	Stomatin‐like protein 2, mitochondrial	Q9UJZ1 (STML2_HUMAN)
*MYCBP2*	E3 ubiquitin‐protein ligase MYCBP2	O75592 (MYCB2_HUMAN)
*ARL6IP6*	ADP‐ribosylation factor‐like protein 6‐interacting protein 6	Q8N6S5 (AR6P6_HUMAN)
*TBCA*	Tubulin‐specific chaperone A	O75347 (TBCA_HUMAN)
*ATE1*	Arginyl‐tRNA‐‐protein transferase 1	O95260 (ATE1_HUMAN)
*RAB10*	Ras‐related protein Rab‐10	P61026 (RAB10_HUMAN)
*RDX*	Radixin	P35241 (RADI_HUMAN)
*MARK2*	Serine/threonine‐protein kinase MARK2	Q7KZI7 (MARK2_HUMAN)
*MARK1*	Serine/threonine‐protein kinase MARK1	Q9P0L2 (MARK1_HUMAN)
*TMED5*	Transmembrane emp24 domain‐containing protein 5	Q9Y3A6 (TMED5_HUMAN)
*DDX21*	Nucleolar RNA helicase 2	Q9NR30 (DDX21_HUMAN)
*ALG5*	Dolichyl‐phosphate beta‐glucosyltransferase	Q9Y673 (ALG5_HUMAN)
*GNG5*	Guanine nucleotide‐binding protein G(I)/G(S)/G(O) subunit gamma 5	P63218 (GBG5_HUMAN)
*HS2ST1*	Heparan sulphate 2‐O‐sulfotransferase 1	Q7LGA3 (HS2ST_HUMAN)
*SBNO1*	Protein strawberry notch homolog 1	A3KN83 (SBNO1_HUMAN)
*SPART*	Spartin	Q8N0X7 (SPART_HUMAN)
*RAB7A*	Ras‐related protein Rab‐7a	P51149 (RAB7A_HUMAN)

### 3.3 Enrichment analysis

#### 3.3.1 *Gene* *annotation and pathway analysis*

**FIGURE 4:**
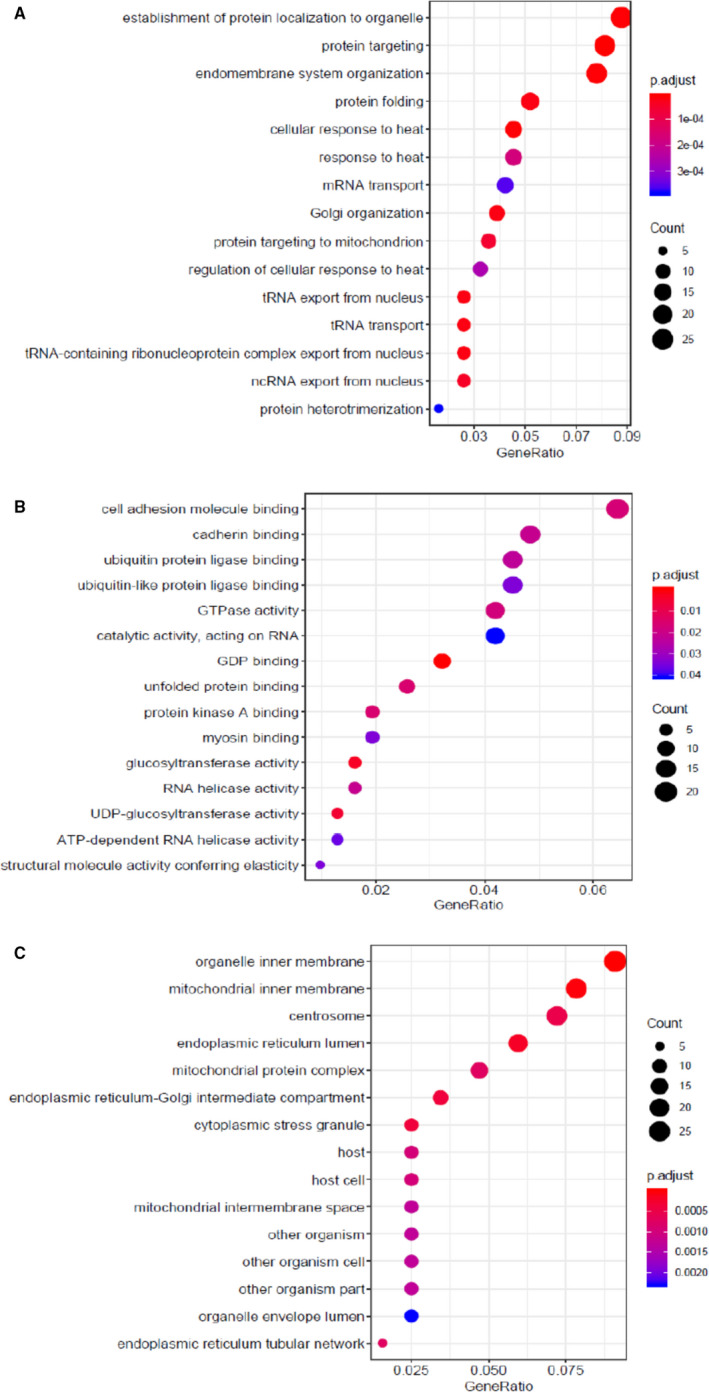
Gene Ontology (GO) enrichment analysis conducted using clusterProfiler R package for 32 target genes related to the respiratory system, cellular and immune pathways, and vitamin D pathways. All GO terms were identified with an adjusted enrichment *P* value of less than .05. A) A bubble plot of the enriched Biological Process (BP), B) a bubble plot of the enriched Molecular Function (MF), and C) a bubble plot of the enriched Cellular Component (CC). The size and colour of the nodes represent the gene number and the p. adjust of each GO terms, respectively

For investigating the relevance of the target genes, these genes were grouped into GO terms. A total of 278 GO categories were significantly enriched including 189 terms of biological processes (BP), 20 molecular function (MF) terms and 69 terms of cellular components (CC). The most important GO terms are shown in Figure 4. The most highly significant GO categories within BP terms were identified as protein targeting, heat response and establishment of protein localization to organelle (Figure 4A). In addition, regulation of cell cycle G2/M phase transition term was also overrepresented (supplementary data file 3). The significantly enriched MF terms were guanosine diphosphate (GDP) binding and glucosyltransferase activity (Figure 4B). In the category of CC, organelle inner membrane, mitochondrial inner membrane and endoplasmic reticulum lumen were the top GO terms (Figure 4C). The pathway analysis also revealed that target genes were enriched only in protein processing in endoplasmic reticulum and RNA transport pathways.

#### 3.3.2 *Identification* *of promoter motifs*

**FIGURE 5:**
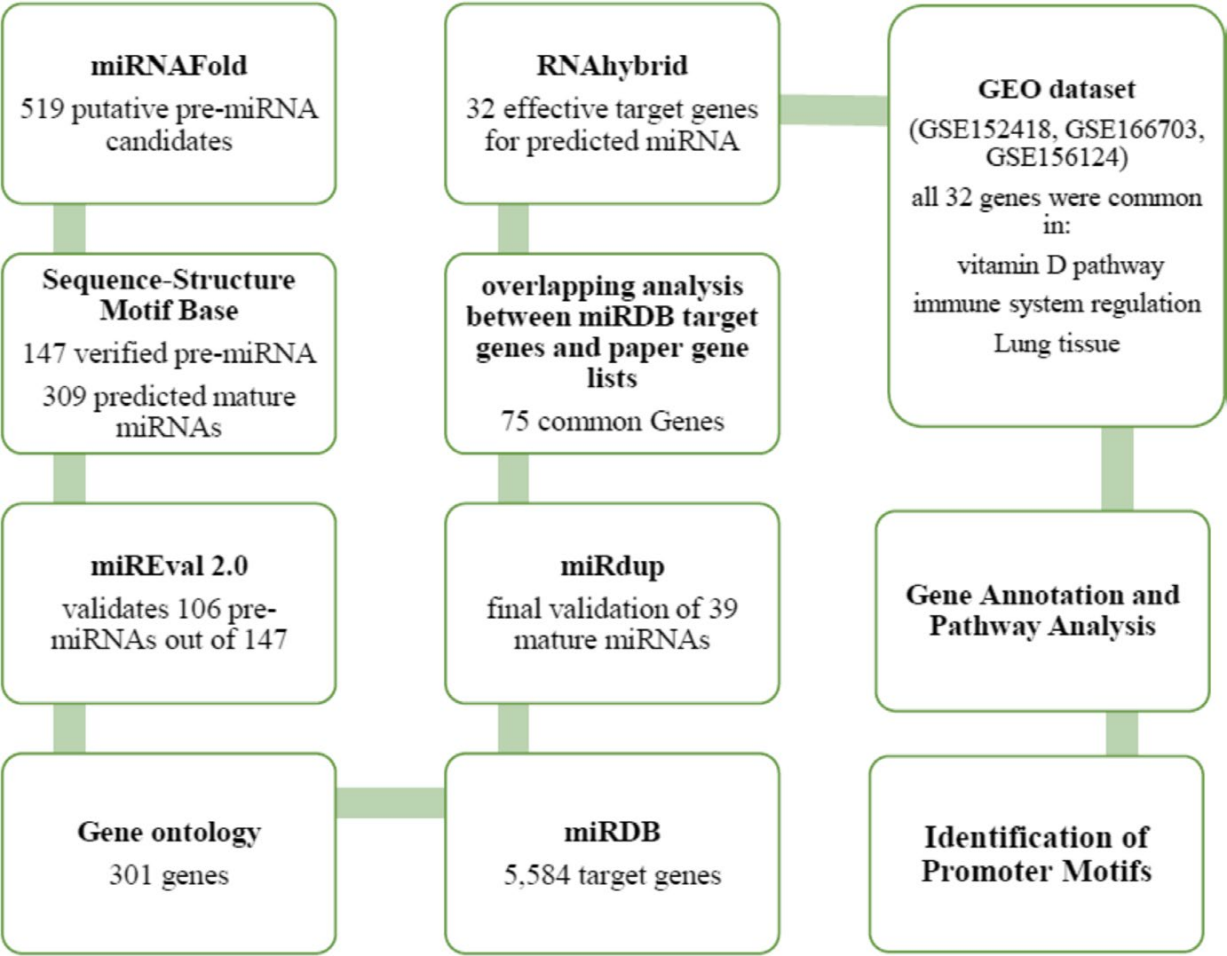
The flow chart of results used in the study

The 1000 bp of 5' regulatory region of target genes was scanned to identify the conserved motifs and consensus CREs. The top 10 significant motifs were discovered by MEME Suite, and motif‐based sequence analysis tools, with widths ranging from 15 to 50aa in the target genes' promoter. GoMo tool predicted several interesting biological processes by further analysing the identified motifs using MEME tool (Table 3). The flow chart of results is provided in Figure 5.

**TABLE 3:** The conserved motifs identified in the promoter region of SARS‐CoV‐2 virus miRNA target genes by MEME analysis and Significant Biological Process identified by UniProtKB Database

	Motif Logo	Width	E. value	Best match in JASPAR and PLACE	Significant GO term identified by GOMO	Significant Biological Process term identified by UniProtKB Database
Motif 1	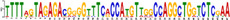	50	4.37e‐04	ZN121_HUMAN.H11MO.0.C	BP Sensory perception of smell BP DNA damage checkpoint	Sensory perception of smell DNA damage checkpoint Cytokine‐mediated signalling pathway inhibitory effect on monocyte differentiation enhancing endopeptidase activity in apoptotic process G1/S transition of mitotic cell cycle Response to drug Response to growth factor
			3.87e‐02	PAX5_HUMAN.H11MO.0.A	BP Regulation of B cell receptor signalling pathway BP Humoral immune response	Regulating B cell receptor signalling pathway Humoral immune response
Motif 2	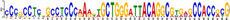	50	‐	‐	BP Nuclear mRNA splicing, via spliceosome BP Translational elongation	_
Motif 3	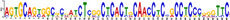	50	‐	‐	BP Positive regulation of transcription from RNA polymerase II promoter BP Small GTPase mediated signal transduction	_
Motif 4	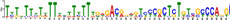	50	2.99e‐05	PAX5_HUMAN.H11MO.0.A	BP Sensory perception of smell BP RNA splicing BP Translational elongation	Regulation of B cell receptor signalling pathway Humoral immune response
			3.14e‐04	ZN121_HUMAN.H11MO.0.C		Sensory perception of smell DNA damage checkpoint Cytokine‐mediated signalling pathway Inhibitory effect on monocyte differentiation enhancing endopeptidase activity involved in apoptotic process G1/S transition of mitotic cell cycle Response to drug Response to growth factor
Motif 5	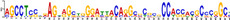	50	‐	‐	BP Chromatin modification	_
Motif 6	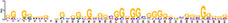	41	1.94e‐08	SP2_HUMAN.H11MO.0.A	BP Negative regulation of signal transduction BP Inner ear morphogenesis BP Actin cytoskeleton organization	Immune response
			1.04e‐07	PATZ1_HUMAN.H11MO.0.C		T cell differentiation
Motif 7	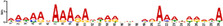	29	1.94e‐02	SPIB_HUMAN.H11MO.0.A	BP Sensory perception of smell BP G protein–coupled receptor protein signalling pathway BP Regulation of immune response BP Transcription initiation from RNA polymerase II promoter	Cell differentiation
			1.01e‐02	PRDM6_HUMAN.H11MO.0.C		Regulation of gene expression Negative regulation of transcription by RNA polymerase II
Motif 8	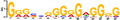	15	2.10e‐04	VEZF1_HUMAN.H11MO.0.C	BP Interior/posterior pattern formation BP Negative regulation of signal transduction	Cellular defence response Endothelial cell development
			1.28e‐03	ZBT17_HUMAN.H11MO.0.A		IRE1‐mediated unfolded protein response Positive regulation of cell cycle arrest
Motif 9	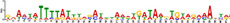	42	1.26e‐02	Z354A_HUMAN.H11MO.0.C	BP Sensory perception of smell BP G protein–coupled receptor protein signalling pathway BP positive regulation of immune response BP defence response	Response to folic acid Response to hypoxia
Motif 10	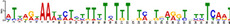	41	1.70e‐02	PRDM6_HUMAN.H11MO.0.C	BP sensory perception of smell BP G protein–coupled receptor protein signalling pathway BP defence response BP positive regulation of immune response	Regulation of gene expression Negative regulation of transcription by RNA polymerase II

## 4 DISCUSSION

SARS‐CoV‐2 has infected about 53 million people worldwide until now, of which approximately 929,000 people have died, making it a global crisis. Despite successful achievements in sequencing of the virus, which has greatly helped to understand origin of the virus and its infectivity unfortunately, complexity of the virus and its variable behaviour have prevented full mastery and recognition of its pathogenicity. Consequently, efforts for treating or finding a vaccine have proceeded slowly so far.^3^

With the advent of microRNAs into the world of molecular biology, these powerful molecules were soon able to make their way into studies in the field of disease genetics.^40^

Some studies have investigated the role of miRNA in COVID‐19 infection. Anti‐viral miRNAs in the host cells can be crucial during viral infections. The miRNAs can be considered as a potential therapeutic molecule.^41^, ^42^

Two different methods are used to detect v‐miRNAs. In the first method, secondary structures of the virus and finally viral miRNAs are predicted using bioinformatics methods and through the virus genome. Despite the possible errors and false‐positive results, this method has greatly contributed in predicting viral miRNAs. Moreover, rate of false‐positive results can be decreased by applying multiple tools. The second method is using sequencing for small RNA clones.^43^, ^44^

In the present study, one of the possible pathogenic aspects of SARS‐CoV‐2 was investigated based on microRNAs encoded by the virus, which may act in altering expression of key genes in important biological pathways including the humans҆ lung tissue cells, vitamin D and inflammation to determine whether the virus miRNAs could exert significant role in pathogenicity of the virus. These biological pathways seem to be among the most important targets of SARS‐CoV‐2. For investigating this process, viral miRNAs and human target genes were studied and predicted to determine whether the SARS‐CoV‐2 miRNAs could play a significant role in the virus's pathogenicity.

Using bioinformatics tools, each algorithm has a special rate of false‐positive and false‐negative results.^45^ Therefore, exploiting more than one algorithm is necessary to make reliable predictions about miRNAs and target genes. Here, miRNAFold, RNAfold, PriMir, Mirident miREval 2.0 and miRdup software were used for prediction of miRNA, and miRDB database, Venn diagram and RNAhybrid tool were applied for prediction of target genes.

Functional analysis revealed that two genes of G protein subunit gamma 5(GNG5) and fibrillin 2(FBN2) were highly down‐regulated and associated with signal transduction and transforming growth factor beta (TGF**‐** *β*) pathway, respectively. G proteins are involved in various transmembrane signalling systems. Most of the subunits, such as GNG5, are expressed on the immune cells' surface. Regulation of many immune functions is associated with G proteins. They are involved in activation, migration, proliferation and cytokine secretion of immune cells.^39^ On the other hand, fibrillin could potently regulate many pathways of the immune response, inflammation and storage and bioavailability of TGF‐β, as a cytokine with a dual role in both inflammatory and suppressive immune responses. The effect of miRNA on fibrillin expression has been approved previously. The microRNAs are involved in disorders caused by fibrillin deficiency.^46^ It has been also clarified that some of human key genes, such as integrin subunit beta 1(ITGB1), ribonucleic acid export 1(RAE1), Dexd‐box helicase 21(DDX21) and Ras‐related protein Rab‐7a (RAB7A) play a role in virus pathogenesis, for example in virus receptor activity, intracellular transport of virus and defence response to the virus and participating in life cycle of viruses, respectively.

Results of a recent in vitro study confirmed that Ebola virus (EBOV)–derived pre‐miRNAs are dependently processed by cellular miRNA processing machinery into subsequent mature miRNAs that like host miRNAs can directly silence their target mRNA.^47^ Islam et al predicted several novel miRNAs produced by Zika virus. They showed that one of the most important Zika virus pathogeneses might be caused by viral microRNAs. These miRNAs target the genes associated with cellular immunity and neurogenic functions.^47^ Interestingly, results of two experimental studies, in which tick‐borne encephalitis virus (TBEV) and Sindbis virus were artificially modified by inserting an exogenous miRNA hairpin, confirmed that cytoplasmic RNA virus could express functional miRNA motifs, for example it was discovered that KUN‐miR‐1, produced by West Nile RNA virus, targets GATA binding protein 4(GATA4) and leads to virus replication.^48^, ^49^ In another study, it was shown that Dengue virus miRNA ‘DENV–vsRNA‐5’ targets non‐structural protein 1(NS1) and has a role in autoregulation of the virus.^50^

Since transcription factors (TFs) play a role in immune response, vitamin D pathway and the lung tissue cells, a precise annotation of human TFs seems to be essential to improve our understanding of their molecular features and functions in SARS‐CoV‐2 pathogenesis. Also, according to the existing reports, silencing expression of corresponding or other viral‐encoded miRNAs with single‐stranded complementary oligonucleotides, or so‐called anti‐miRNA oligonucleotides may be a valuable therapeutic tool while combating viral infections and pathogeneses.^51^

In this study, the genes having the most important role in mechanisms of pathogenesis of COVID‐19 were identified. Based on our findings obtained from bioinformatics tools, gene silencing is one of key mechanisms of the SARS‐CoV‐2 virus. Repressing the immune response genes leads to infection and inhibition of the immune response and accelerates the virus's pathogenesis. However, any attempt to pinpoint biogenesis of miRNAs encoded by RNA, their functional mechanism in virus‐host networks and their potential as biomarkers will provide valuable insights in this regard. Results of our study were not validated by experimental analysis, and only bioinformatics evaluations were emphasized. But, reliance on bioinformatics studies alone can lead to deviations in reports. Hence, it is suggested to perform laboratory tests in order to validate results of the present study.

## 5 CONCLUSION

In this study, we investigated viral miRNAs as a principal factor in the pathogenicity of SARS‐CoV‐2. MicroRNAs can act on pathogenesis of the disease by targeting and reducing the expression of key genes involved in the immune system against viruses and the pathway of the respiratory system and vitamin D. We have predicted several new microRNAs produced by SARS‐CoV‐2 using bioinformatics tools, and as expected, the target genes of these miRNAs play an important role in lung tissue cells, vitamin D and inflammatory processes.

## CONFLICT OF INTEREST

The authors declare that they have no conflict of interests.

## AUTHOR CONTRIBUTION

**Elham karimi:** Conceptualization (equal); Data curation (equal); Investigation (equal); Writing‐original draft (lead); Writing‐review & editing (lead). **Hanie Azari:** Conceptualization (equal); Data curation (equal); Investigation (equal); Writing‐original draft (lead); Writing‐review & editing (lead). **Maryam Yari:** Writing‐original draft (equal); Writing‐review & editing (equal). **Ahmad Tahmasebi:** Data curation (equal); Writing‐original draft (equal); Writing‐review & editing (supporting). **mehdi hassani azad:** Data curation (supporting); Writing‐review & editing (equal). **Pegah Mousavi:** Conceptualization (lead); Data curation (lead); Investigation (lead); Project administration (lead); Supervision (lead); Writing‐original draft (lead); Writing‐review & editing (lead).

## Supporting information

Supplementary Material

Supplementary Material

Supplementary Material

## Data Availability

Data available on request due to privacy/ethical restrictions.
