# Supplementary material for: Interplay between SARS‐CoV‐2‐derived miRNAs, immune system, vitamin D pathway and respiratory system
Source: J Cell Mol Med. 2021 Jun 22;25(16):7825–39. doi: 10.1111/jcmm.16694 (PMC8358877; doi:10.1111/jcmm.16694)
Supplement: Supplementary file 1 — Supplementary Material [file JCMM-25-7825-s001.docx]

Supplementary data1. Mature miRNAs validated by sequence-structure Motif Base, miREval, and miRdup web server

| **miRNA Name** | **Precursor sequence** | **Hairpin (MFE)** |
| --- | --- | --- |
| SCoV-2-miR-1 | UCACAUCUGAUUUGGCUACUAACAAUCUAGUUGUAAUGGCCUACAUUACAGGUGGUGUUGUUCAGUUGACUUCGCAGUGGGC | ...((.(((....(((.((((((((((((.((((((((.....)))))))).))).)))))).))).).))...)))))... (-23.30) |
| SCoV-2-miR-2 | GGUUGAGCUGGUAGCAGAACUCGAAGGCAUUCAGUACGGUCGUAGUGGUGAGACACUUGGUGUCCUUGUCCCUCAUGUGGGCGAAAUACCAGUGGCUUACCGCAAGGUUCUUCUUCGUAAGAACGGUAAUAAAGGAGCUGGUGGCC | ((((.((((..(...(..(((........(((..(((((.(.(.((((((((....(((((((..((((((.(...).)))))).)))))))...)))))))).).).......))))).))).)))..)..)..))))...))))(-46.90) |
|  | UUGAGCUGGUAGCAGAACUCGAAGGCAUUCAGUACGGUCGUAGUGGUGAGACACUUGGUGUCCUUGUCCCUCAUGUGGGCGAAAUACCAGUGGCUUACCGCAAGGUUCUUCUUCGUAAGAACGGUAAUAAAGGAGCUGGUGGCCAUAG | ....(((((..((...........))..)))))..(((((..((((((((.((((.(((((..((((((.......)))))).))))))))).))))))))..(((((((..((((....))))......)))))))..)))))....(-46) |
| SCoV-2-miR-3 | GCCUUGUCCCUGGUUUCAACGAGAAAACACACGUCCAACUCAGUUUGCCUGUUUUACAGGUUCGCGACGUGCUCGUACGUGGCUUUGGAGACUCCGU | .....(((((.((((...(((((......((((((.......((..(((((.....)))))..)))))))))))))....))))..)).)))..... (-26.61) |
| SCoV-2-miR-4 | UGAGACUCAUUGAUGCUAUGAUGUUCACAUCUGAUUUGGCUACUAACAAUCUAGUUGUAAUGGCCUACAUUACAGGUGGUGUUGUUCAGUUGACUUCGCAGUGGCUAACUAACAUCUUUGGCACUGUUUAUGAAAAACUCA | ((((..((((.(.(((((.((((((..........(((((((((.((((((...((((((((.....))))))))....))))))..............)))))))))..))))))..)))))....).))))....))))(-42.2) |
| SCoV-2-miR-5 | GUUGACUUCGCAGUGGCUAACUAACAUCUUUGGCACUGUUUAUGAAAAACUCAAACCCGUCCUUGAUUGGCUUGAAGAGAAGUUUAAGGAAGGUGUAGAGUUUCUUAGAGACGGUUGGGAAAUUGUUAAAUUUAUCUCAAC | (((((.......((((.(...(((((..(((...(((((((.(((.((((((..(((..(((((((.(..(((...)))..).))))))).)))...)))))).))).)))))))...)))..))))).).)))).)))))(-46.6) |
| SCoV-2-miR-6 | UGAACUUGAUGAAAGGAUUGAUAAAGUACUUAAUGAGAAGUGCUCUGCCUAUACAGUUGAACUCGGUACAGAAGUAAAUGAGUUCGCCUGUGUUGUGGCAGAUGCUGUCAUAAAAACUUUGCAACCAGUAUCUGAAUUACUUA | (((...((((...((.((((.((((((..((.((((...((..((((((.((((((.((((((((.(((....)))..)))))))).))))))...)))))).))..)))).)).))))))....))))..))..)))).)))(-50.7) |
| SCoV-2-miR-7 | UGAACUUGAUGAAAGGAUUGAUAAAGUACUUAAUGAGAAGUGCUCUGCCUAUACAGUUGAACUCGGUACAGAAGUAAAUGAGUUCGCCUGUGUUGUGGCAGAUGCUGUCAUAAAAACUUUGCAACCAGUAUCUGAAUUACUUA | .......((((...((.(((.((((((..((.((((..((((.((((((.((((((.((((((((.(((....)))..)))))))).))))))...)))))))))).)))).)).)))))))))))..))))........... (-50.7) |
|  | UAAAUGAGUUCGCCUGUGUUGUGGCAGAUGCUGUCAUAAAAACUUUGCAACCAGUAUCUGAAUUACUUACACCACUGGGCAUUGAUUUAGAUGAGUGGAGUAUGGCUACAUACUACUUAUUUG | (((((((((......(((.((((((..(((((.((((..(((...((...(((((...((.(.....).))..))))).))....)))..))))....))))).))))))))).))))))))) (-35.9) |
| SCoV-2-miR-8 | CGGCAGUGAGGACAAUCAGACAACUACUAUUCAAACAAUUGUUGAGGUUCAACCUCAAUUAGAGAUGGAACUUACACCAGUUGUUCAGACUAUUGAAGUGAAUAGUUUUAGUGGUUAUUUAAAACUUACUG | (((.(((..(((.(((((...((..(((((((.((((((((.(.((((((.(.(((.....))).).))))))..).))))))))..............))))))).))..))))).)))...)))..))) (-35.2) |
| SCoV-2-miR-9 | UUGAAGUUUAAUCCACCUGCUCUACAAGAUGCUUAUUACAGAGCAAGGGCUGGUGAAGCUGCUAACUUUUGUGCACUUAUCUUAGCCUACUGUAAUAAGACAGUAGGUGAGUUAGGUGAUGUUAG | ((((...(((((.((((((((..........((((((((((.....(((((((...((.(((.((...))..))).))...))))))).))))))))))..)))))))).)))))......)))) (-38.9) |
|  | UGCUUAUUACAGAGCAAGGGCUGGUGAAGCUGCUAACUUUUGUGCACUUAUCUUAGCCUACUGUAAUAAGACAGUAGGUGAGUUAGGUGAUGUUAGAGAAACAAUGAGUUACUUGUUUCAACAUGCCAAUUUAGAUUCUUGCA | (((.......((((.((.(((.(.((((((.(.((((((.(((.((((((.(((.(((((((((......)))))))))))).))))))...........)))..)))))).).)))))).)..)))..))....)))).))) (-50.4) |
| SCoV-2-miR-10 | UUGAAGUUUAAUCCACCUGCUCUACAAGAUGCUUAUUACAGAGCAAGGGCUGGUGAAGCUGCUAACUUUUGUGCACUUAUCUUAGCCUACUGUAAUAAGACAGUAGGUGAGUUAGGUGAUGUUAG | ((((...(((((.((((((((..........((((((((((.....(((((((...((.(((.((...))..))).))...))))))).))))))))))..)))))))).)))))......)))) (-38.9) |
|  | UGCUUAUUACAGAGCAAGGGCUGGUGAAGCUGCUAACUUUUGUGCACUUAUCUUAGCCUACUGUAAUAAGACAGUAGGUGAGUUAGGUGAUGUUAGAGAAACAAUGAGUUACUUGUUUCAACAUGCCAAUUUAGAUUCUUGCA | (((.......((((.((.(((.(.((((((.(.((((((.(((.((((((.(((.(((((((((......)))))))))))).))))))...........)))..)))))).).)))))).)..)))..))....)))).))) (-50.4) |
| SCoV-2-miR-11 | UGCUUAUUACAGAGCAAGGGCUGGUGAAGCUGCUAACUUUUGUGCACUUAUCUUAGCCUACUGUAAUAAGACAGUAGGUGAGUUAGGUGAUGUUAGAGAAACAAUGAGUUACUUGUUUCAACAUGCCAAUUUAGAUUCUUGCA | (((.......((((.((.(((.(.((((((.(.((((((.(((.((((((.(((.(((((((((......)))))))))))).))))))...........)))..)))))).).)))))).)..)))..))....)))).))) (-50.4) |
|  | UUGAAGUUUAAUCCACCUGCUCUACAAGAUGCUUAUUACAGAGCAAGGGCUGGUGAAGCUGCUAACUUUUGUGCACUUAUCUUAGCCUACUGUAAUAAGACAGUAGGUGAGUUAGGUGAUGUUAG | ((((...(((((.((((((((..........((((((((((.....(((((((...((.(((.((...))..))).))...))))))).))))))))))..)))))))).)))))......))))  (-38.9) |
| SCoV-2-miR-12 | UGCUUAUUACAGAGCAAGGGCUGGUGAAGCUGCUAACUUUUGUGCACUUAUCUUAGCCUACUGUAAUAAGACAGUAGGUGAGUUAGGUGAUGUUAGAGAAACAAUGAGUUACUUGUUUCAACAUGCCAAUUUAGAUUCUUGCA | (((.......((((.((.(((.(.((((((.(.((((((.(((.((((((.(((.(((((((((......)))))))))))).))))))...........)))..)))))).).)))))).)..)))..))....)))).))) (-50.4) |
|  | GACAGUAGGUGAGUUAGGUGAUGUUAGAGAAACAAUGAGUUACUUGUUUCAACAUGCCAAUUUAGAUUCUUGCA | ....(((((((((((.(((.(((((...(((((((........)))))))))))))))))))))....))))). (-17.20) |
| SCoV-2-miR-13 | UUACUAGAAGUACAAAUUCUAGAAUUAAAGCAUCUAUGCCGACUACUAUAGCAAAGAAUACUGUUAAGAGUGUCGGUAAAUUUUGUCUAGAGGCUUCAUUUAAUUAUUUGAAGUCACCUAA | (((...((..(.(((((......(((((((..((((.(((((((..(.(((((........))))).)..)))))))..........))))......))))))).))))).).))...))) (-26.6) |
| SCoV-2-miR-14 | GAUACAUUCUGUGCUGGUAGUACAUUUAUUAGUGAUGAAGUUGCGAGAGACUUGUCACUACAGUUUAAAAGACCAAUAAAUCCUACUGACCAGUCUUCUUACAUCGUUGAUAGUGUUACAGUGAAGAAUGGUUC | ((...((((((..(((.....(((((..(.....((((.((.(...((((((.((((.((..(((((.........)))))..)).)))).)))))).).)).)))).)..)))))..)))..))))))...)) (-40) |
| SCoV-2-miR-15 | GAUACAUUCUGUGCUGGUAGUACAUUUAUUAGUGAUGAAGUUGCGAGAGACUUGUCACUACAGUUUAAAAGACCAAUAAAUCCUACUGACCAGUCUUCUUACAUCGUUGAUAGUGUUACAGUGAAGAAUGGUUC | ((...((((((..(((.....(((((..(.....((((.((.(...((((((.((((.((..(((((.........)))))..)).)))).)))))).).)).)))).)..)))))..)))..))))))...)) (-40) |
|  | AGUCUUCUUACAUCGUUGAUAGUGUUACAGUGAAGAAUGGUUCCAUCCAUCUUUACUUUGAUAAAGCUGGUCAAAAGACU | (((((..........(((((..(((((.((((((((..(((...)))..)))))))).)))))......))))).))))) (-21.7) |
| SCoV-2-miR-16 | AGUCUUCUUACAUCGUUGAUAGUGUUACAGUGAAGAAUGGUUCCAUCCAUCUUUACUUUGAUAAAGCUGGUCAAAAGACU | (((((..........(((((..(((((.((((((((..(((...)))..)))))))).)))))......))))).))))) (-21.7) |
| SCoV-2-miR-17 | CUUUUGAAGAAGCUGCGCUGUGCACCUUUUUGUUAAAUAAAGAAAUGUAUCUAAAGUUGCGUAGUGAUGUGCUAUUACCUCUUACGCAAUAUAAUAGAUACUUAGCUCUUUAUAAUAAGUACAAGUAUUUUAGUGGAGCAAUGG | ((.(((.....((((....((((...(.((((((...(((((((..(((((((..(((((((((.(..(((....))).).)))))))))....)))))))....))))))).)))))).)...))))..))))....))).)) (-41.3) |
| a | CUCUGAAGACAUGCUUAACCCUAAUUAUGAAGAUUUACUCAUUCGUAAGUCUAAUCAUAAUUUCUUGGUACAGGCUGGUAAUGUUCAACUCAGGG | ((((((.(((((((((.(((..((((((((((((((((......))))))))..))))))))....)))..)))).....)))))....)))))) (-25.50) |
| SCoV-2-miR-19 | UUCAUUCCUUAAUGGUUCAUGUGGUAGUGUUGGUUUUAACAUAGAUUAUGACUGUGUCUCUUUUUGUUACAUGCACCAUAUGGAAUUACCA | ...(((((...(((((.(((((..(((....((.....((((((.......))))))..))..)))..))))).)))))..)))))..... (-22.60) |
| SCoV-2-miR-20 | UUACUGCAAAAUGGUAUGAAUGGACGUACCAUAUUGGGUAGUGCUUUAUUAGAAGAUGAAUUUACACCUUUUGAUGUUGUUAGACAAUGCUCAGGUGUUACUUUCCAAAGUGCAGUGA | ((((((((............((((.(((.(((..((((((..((..(((((((((.((......)).)))))))))..)).......)))))).))).)))..))))...)))))))) (-39.2) |
| SCoV-2-miR-21 | UUUUAAUAUGGUCUAUAUGCCUGCUAGUUGGGUGAUGCGUAUUAUGACAUGGUUGGAUAUGGUUGAUACUAGUUUGUCUGGUUUUAAGCUAAAAGACUGUGUUAUGUAUGCAUCAGCUGUAGUGUUACUAAUCCUUAUGA | ((.(((..((((........((((......(.((((((((((.(((((((.(((...(.(((((.(.(((((.....)))))..).))))).).))).))))))))))))))))).).))))....))))....))).)) (-41.8) |
| SCoV-2-miR-22 | CAUAUAAAAAUACGUGUGAUGGUACAACAUUUACUUAUGCAUCAGCAUUGUGGGAAAUCCAACAGGUUGUAGAUGCAGAUAGUAAAAUUGUUCAACUUAGUGAAAUUAGUAUG | (((((.((..(((.......(((..((((.((((((.((((((.(((.(.((((...))))....).))).))))))..))))))...))))..)))..)))...)).))))) (-33.9) |
| SCoV-2-miR-23 | UGGUACUGGUCAGGCAAUAACAGUUACACCGGAAGCCAAUAUGGAUCAAGAAUCCUUUGGUGGUGCAUCGUGUUGUCUGUACUGCCGUUGCCACAUAGAUCAUCCA | (((...(((((.((((((..((((.....(((....((((((((..((...(((....)))..))..)))))))).))).))))..)))))).....))))).))) (-34.4) |
| SCoV-2-miR-24 | AUGCCUAUAUUAACCUUGACCAGGGCUUUAACUGCAGAGUCACAUGUUGACACUGACUUAACAAAGCCUUACAUUAAGUGGGAUUUGUUAAAAUAUGACUUCACGGAAGAGAGGUUAAAACUCUUUGACCGUUAUUUUAAAUAU | (((..((.(.((((..(.(..((((.(((((((.(.((((((.((.((((((.(.((((((............)))))).)....)))))).)).)))))).........).))))))).)))).).)..)))).).))..))) (-39.3) |
|  | AUUCAUUGUUAAUGCCUAUAUUAACCUUGACCAGGGCUUUAACUGCAGAGUCACAUGUUGACACUGACUUAACAAAGCCUUACAUUAAGUGGGAUUUGUUAAAAUAUGACUUCACGGAAGAGAGGUUAAAACUCUUUGACCGUUAU | ((..((.(((((.(..(...((((((((........((((.......((((((.((.((((((.(.((((((............)))))).)....)))))).)).))))))....)))).)))))))).)..).))))).)).)) (-37.2) |
| SCoV-2-miR-25 | AUGCCUAUAUUAACCUUGACCAGGGCUUUAACUGCAGAGUCACAUGUUGACACUGACUUAACAAAGCCUUACAUUAAGUGGGAUUUGUUAAAAUAUGACUUCACGGAAGAGAGGUUAAAACUCUUUGACCGUUAUUUUAAAUAU | ((..((.(((((.(..(...((((((((........((((.......((((((.((.((((((.(.((((((............)))))).)....)))))).)).))))))....)))).)))))))).)..).))))).)).)) (-39.3) |
|  | AUUCAUUGUUAAUGCCUAUAUUAACCUUGACCAGGGCUUUAACUGCAGAGUCACAUGUUGACACUGACUUAACAAAGCCUUACAUUAAGUGGGAUUUGUUAAAAUAUGACUUCACGGAAGAGAGGUUAAAACUCUUUGACCGUUAU | ((..((.(((((.(..(...((((((((........((((.......((((((.((.((((((.(.((((((............)))))).)....)))))).)).))))))....)))).)))))))).)..).))))).)).)) (-37.2) |
| SCoV-2-miR-26 | ACUACUAUUUGUAGUUGAAGUUGUUGAUAAGUACUUUGAUUGUUACGAUGGUGGCUGUAUUAAUGCUAACCAAGUCAUCGUCAACAACCUAGACAAAUCAGCUGGUUUUCCAUUUAAUAAAUGGGGU | ((..((((((((.((.((((..((((((..((...((..((((...((((((((((...(((....)))...)))))))))).))))..)).))..))))))....)))).))...)))))))).)) (-38) |
| SCoV-2-miR-27 | GAGCCAUGCCUAACAUGCUUAGAAUUAUGGCCUCACUUGUUCUUGCUCGCAAACAUACAACGUGUUGUAGCUUGUCACACCGUUUCUAUAGAUUAGCUAAUGAGUGUGCUCAAGUAUUGAGUGAAAUGGUCAUGUGUGGCGGUUC | (((((..((((.(((((.......((((....(((((.((.((((..((((..(((.....((.((((((..((......))...))))))....))..)))..))))..)))).))..))))).)))).))))).))))))))) (-19.80) |
| SCoV-2-miR-28 | UCUGCUCGCAUAGUGUAUACAGCUUGCUCUCAUGCCGCUGUUGAUGCACUAUGUGAGAAGG | (((.((((((((((((((.(((((.((......)))))))...)))))))))))))).))) (-29.6) |
| SCoV-2-miR-29 | GGAACUUGUCGGCGUUGUCCUGCUGAAAUUGUUGACACUGUGAGUGCUUUGGUUUAUGAUAAUAAGCUUAAAGCACAUAAAGACAAAUCAGCUCAAUGCUUUAAAAUGUUUU | (((((.((..(((((((....(((((..(((((.......((.(((((((((.((.........)).))))))))).))..))))).))))).)))))))..))...))))) (-36.8) |
| SCoV-2-miR-30 | GCAUCAUUCUAUUGGAUUUGAUUACGUCUAUAAUCCGUUUAUGAUUGAUGUUCAACAAUGGGGUUUUACAGGUAACCUACAAAGCAACCAUGAUCUGUAUUGUCAAGUCCAUGGUAAUGCACAUGUAGCUAGUUGUGAUGC | (((((((...(((((.......((((((..((..((((..((...((((...((...((((.(((((..(((...)))..)))))..))))....))....)))).))..))))...))..)))))).))))).))))))) (-39.1) |
| SCoV-2-miR-31 | UGUGGGUUUAUACAACAAAAGCUAGCUCUUGGAGGUUCCGUGGCUAUAAAGAUAACAGAACAUUCUUGGAAUGCUGAUCUUUAUAAGCUCAUGGGACACUUCGCAUGGUGGACAGCCUUUGUUACUAAUGUGAAUGCGUCA | ((.(.(((((((.((((((.(((.(((..((((((((((((((.(((((((((.(.....(((((...))))).).)))))))))...))))))))).)))))...)))....))).)))))).....))))))).)..)) (-45.8) |
| SCoV-2-miR-32 | AACCUUUUGAGAGAGAUAUUUCAACUGAAAUCUAUCAGGCCGGUAGCACACCUUGUAAUGGUGUUGAAGGUUUUAAUUGUUACUUUCCUUUACAAUCAUAUGGUU | (((((..((.(((((......(((.((((((((.(((.((((...(((.....)))..))))..))))))))))).)))...))))).........))..))))) (-27.9) |
| SCoV-2-miR-33 | GUGCAGGUAUAUGCGCUAGUUAUCAGACUCAGACUAAUUCUCCUCGGCGGGCACGUAGUGUAGCUAGUCAAUCCAUCAUUGCCUACACUAUGUCACUUGGUGCAGAAAAUUCAGUUGCUUACUCUAAUAACUCUAUUGCCAUACCCAC | ((...(((((..(((..((((((.(((.(..((((..((((...((.((((.(((((((((((.((((.(.....).)))).)))))))))))..)))).)).)))).....))))....).))).))))))....))).))))).)) (-51.4) |
| SCoV-2-miR-34 | GUGCAGGUAUAUGCGCUAGUUAUCAGACUCAGACUAAUUCUCCUCGGCGGGCACGUAGUGUAGCUAGUCAAUCCAUCAUUGCCUACACUAUGUCACUUGGUGCAGAAAAUUCAGUUGCUUACUCUAAUAACUCUAUUGCCAUACCCAC | ((...(((((..(((..((((((.(((.(..((((..((((...((.((((.(((((((((((.((((.(.....).)))).)))))))))))..)))).)).)))).....))))....).))).))))))....))).))))).)) (-51.4) |
| SCoV-2-miR-35 | CAUGGUACAUUUGGCUAGGUUUUAUAGCUGGCUUGAUUGCCAUAGUAAUGGUGACAAUUAUGCUUUGCUGUAUGACCAGUUGCUGUAGUUGUCUCAAGGGCUGUUGUUCUUGUGGAUCCUGCUGCAAAUUUGAUG | ((...((.(((((..((((.(((((((...........((.(((((..((..((((((((.((...((((......)))).))..)))))))).))...))))).))..))))))).))))...))))).)).)) (-37.8) |
| SCoV-2-miR-36 | GUAUUGCUGGACACCAUCUAGGACGCUGUGACAUCAAGGACCUGCCUAAAGAAAUCACUGUUGCUACAUCACGAACGCUUUCUUAUUACAAAUUGGGAGCUUCGCAGCGUGUAGCAGGUGACUCAGGUUUUGC | (((...((((.((((..(((..(((((((((..((((.....((....((((((....((((..(.....)..)))).))))))....))..)))).....))))))))).)))..))))..))))....))) (-42.5) |
| SCoV-2-miR-37 | GGUUGAGCUGGUAGCAGAACUCGAAGGCAUUCAGUACGGUCGUAGUGGUGAGACACUUGGUGUCCUUGUCCCUCAUGUGGGCGAAAUACCAGUGGCUUACCGCAAGGUUCUUCUUCGUAAGAACGGUAAUAAAGGAGCUGGUGGCC | ((((.((((..(...(..(((........(((..(((((.(.(.((((((((....(((((((..((((((.(...).)))))).)))))))...)))))))).).).......))))).))).)))..)..)..))))...)))) (-46.9) |
|  | UUGAGCUGGUAGCAGAACUCGAAGGCAUUCAGUACGGUCGUAGUGGUGAGACACUUGGUGUCCUUGUCCCUCAUGUGGGCGAAAUACCAGUGGCUUACCGCAAGGUUCUUCUUCGUAAGAACGGUAAUAAAGGAGCUGGUGGCCAUAG | (((.((...((((....((..(..((.(((..(((((.(.(.((((((((....(((((((..((((((.(...).)))))).)))))))...)))))))).).).......))))).)))..))..)..))..))))...))..))) (-46) |
| SCoV-2-miR-38 | CUCUGAAGACAUGCUUAACCCUAAUUAUGAAGAUUUACUCAUUCGUAAGUCUAAUCAUAAUUUCUUGGUACAGGCUGGUAAUGUUCAACUCAGGG | ((((((.(((((..(((..(((...(((.((((.(((...(((.(.....).)))..)))..)))).))).))).)))..)))))....)))))) (-26.4) |
| SCoV-2-miR-39 | GUCGUAGUGGUGAGACACUUGGUGUCCUUGUCCCUCAUGUGGGCGAAAUACCAGUGGCUUACCGCAAGGUUCUUCUUCGUAAGAACGGUAAUAAAGGAGCUGGUGGCCAUAGUUACGGCGCCGAUCUAAAGUCAUUUGACUUAGGCGAC | (((((...(((.(((.((((.(.(((...((((....(((((......(((((((...((((((.........((((...)))).)))))).......))))))).)))))....))))...))).).))))..))).)))...))))) (-46.9) |
